# FAST technique: fast atrial sheath traction technique for device closure of atrial septal defects

**DOI:** 10.3389/fcvm.2023.1155142

**Published:** 2023-05-22

**Authors:** Raymond N. Haddad, Rachid Kaddoura, Mohamed Kasem, Mahmoud Alsoufi

**Affiliations:** ^1^ Centre de Référence Malformations Cardiaques Congénitales Complexes—M3C, Hôpital Universitaire Necker-Enfants Malades, Assistance Publique—Hôpitaux de Paris, Paris, France; ^2^ College of Medicine, Mohammed Bin Rashid University of Medicine and Health Sciences, Dubai, United Arab Emirates; ^3^ Department of Pediatric Cardiology, Heart Centre of Excellence, Al Jalila Children's Speciality Hospital, Dubai, United Arab Emirates

**Keywords:** atrial septal defect, device closure, innovation, modified technique, transcatheter interventions

## Abstract

**Background:**

Transcatheter closure of atrial septal defects (ASDs) is well-established. However, this procedure can be challenging, requiring multiple attempts and advanced implantation maneuvers.

**Materials and methods:**

From July 2019 to July 2022, patients to whom the fast atrial sheath traction (FAST) technique was applied for ASD device closure were prospectively followed up. The device was rapidly unsheathed in the middle of the left atrium (LA) to let it clamp the ASD from both sides simultaneously. This novel technique was directly applied in patients with absent aortic rims and/or ASD size-to-body weight ratio higher than 0.9 or after failed attempts of standard implantation.

**Results:**

Seventeen patients (64.7% males) were involved with a median age of 9.8 years [interquartile range (IQR), 7.6–15.1] and a median weight of 34 kg (IQR, 22–44). The median ASD size on ultrasound was 19 mm (IQR, 16–22). Five (29.4%) patients had absent aortic rims, and three (17.6%) patients had an ASD size-to-body weight ratio higher than 0.9. The median device size was 22 mm (IQR, 17–24). The median difference between device size and ASD two-dimensional static diameter was 3 mm (IQR, 1–3). All interventions were straightforward without any complications using three different occluder devices. One device was removed before release and upsized to the next size. The median fluoroscopy time was 4.1 min (IQR, 3.6–4.6). All patients were discharged the next postoperative day. On a median follow-up of 13 months (IQR, 8–13), no complications were detected. All patients achieved full clinical recovery with complete shunt closure.

**Conclusion:**

We present a new implantation technique to efficiently close simple and complex ASDs. The FAST technique can be of benefit in overcoming left disc malalignment to the septum in defects with absent aortic rims and in avoiding complex implantation maneuvers and the risks of injuring the pulmonary veins.

## 1. Introduction

Transcatheter device closure of secundum atrial septal defects (ASDs) is a well-established intervention in both pediatric and adult populations (1–3). However, this procedure can sometimes be challenging and requires multiple attempts, longer procedural time, and higher radiation exposure (4–6). Advanced technical deployment maneuvers were reported to overcome these device implantation challenges but can be associated with higher risks of complications (7–13). We found a new device implantation technique for ASD closure. The device is rapidly unsheathed in the middle of the LA to let it clamp the ASD from both sides simultaneously. In this study, we report the safety, efficacy, and outcomes of this novel fast atrial sheath traction (FAST) technique for the closure of simple and complex ASDs.

## 2. Materials and methods

### 2.1. Study design

From July 2019 to July 2022, 68 patients were sent for an attempted transcatheter closure of an ostium secundum ASD at our institution. Approval from a multidisciplinary team was obtained for each patient. Patients to whom we applied the new FAST technique for ASD device closure were included in this study and prospectively followed up. We applied the FAST technique in cases where we failed the first attempt using the standard implantation method. Furthermore, the FAST technique was directly used in patients with absent aortic rims and/or ASD size-to-body weight ratio higher than 0.9. All procedures contributing to this work comply with the ethical standards of the relevant national guidelines on human experimentation and with the Declaration of Helsinki 1975, as was revised in 2008. Approval from the Institutional Review Board was obtained. Written informed consent to perform the procedure and to use the patients’ clinical records was provided by their legal guardians. Baseline to the latest follow-up, clinical data were collected and comprehensively analyzed.

### 2.2. Interventional procedure

All interventions were performed in the catheterization laboratory under general anesthesia, fluoroscopy, and transthoracic or transesophageal echocardiography guidance. Access to the right femoral vein was obtained under ultrasound guidance. Intravenous antibiotics and heparin (100 UI/kg) were administered, and activated clotting time was monitored. Right cardiac catheterization was performed for baseline hemodynamic assessment. The anatomy and size of ASD were assessed on ultrasound without balloon sizing. Closure of the defects were performed using the available self-centering device with a diameter equal to or the next size up from the largest measured defect diameter on two-dimensional ultrasound. The devices available in our armamentarium during the study period were Amplatzer™ Septal Occluder (AGA Medical Corp., USA), Flex II ASD Occluder (Occlutech, Germany), and MemoPart™ ASD Occluder (Lepu Medical, China). We used one extra size for defects with absent aortic rims and the MemoPart devices. In all cases, we always kept the device size-to-ASD static two-dimensional diameter ratio close to 1, and the difference between the device size and the ASD static two-dimensional diameter was ≤4 mm (14). The atrial hole was crossed using a 5-Fr multipurpose diagnostic catheter (Cordis Corp., USA), and a 0.035-in./180-cm-long Amplatz Super Stiff^™^ exchange wire (Boston Scientific Corp., USA) was parked in the left upper pulmonary vein (PV). A short introducer was replaced with an appropriately sized delivery sheath of the device. The delivery sheath was placed in the LA, and the device was kept inside at the tip of the sheath. We made sure that the tip of the sheath is in the middle of the LA just in the direction of the left upper PV. We kept the center of the device at the atrial septum just right to the mid-spinal line. In one single move, we then rapidly unsheathe both left and right retention discs. The sudden move will deploy both discs together, and they will clamp on the defect almost simultaneously (Supplementary Videos S1–S3). In case the alignment of the center is not obtained when the device is all inside the sheath, we protruded part of the left retention disc out of the sheath to form a tiny bulb in the LA and not inside the left upper PV. This maneuver is of benefit in adjusting the position of the center of the device to keep it aligned with the level of the defect (Supplementary Video S4). A stability check was performed under fluoroscopy and ultrasound before its release (Supplementary Videos S5–S9). The implantation procedure was declared successful only when the device was stably implanted into position until hospital discharge.

### 2.3. Follow-up protocol

All patients were kept overnight and were prescribed daily acetylsalicylic acid and bacterial endocarditis prophylaxis. Outpatient follow-up visits were scheduled for 1 week; then 1, 3, 6, and 12 months after the procedure; and thereafter twice a year until 3 years post-device. At the 6-month follow-up, acetylsalicylic acid therapy and bacterial endocarditis prophylaxis were discontinued in patients with complete closure.

### 2.4. Statistical analyses

Categorical variables were reported as frequency and percentage, and continuous variables were represented as median with interquartile range (IQR).

## 3. Results

### 3.1. Demographics

During the study period, 17 patients (64.7% males) were involved. The median age of the patients was 9.8 years (IQR, 7.6–15.1), and their median weight was 34 kg (IQR, 22–44). The patients' demographics are detailed in Table 1.

**Table 1:** Clinical and procedural characteristics of the patients.

CN	Gender	AAI	W	H	CHD/comorbidities	ASD size	AWR	Aortic rim	Qp/Qs	MPAP	SS	Device	Device size	PT	FT	FU
1	F	9.8	33	138	ASD OS	22	0.7	Present	2.6	16	10	ASO	24	13	4.4	36
2	F	5.6	25	129	ASD OS	18	0.7	Present	1.9	18	8	ASO	19	12	4.1	36
3	F	59.9	68	158	ASD OS/chronic AF	24	0.4	Absent	2.1	32	12	FAO	28	11	4.3	28
4	M	9.2	41	142	ASD OS	16	0.4	Present	1.8	16	8	ASO	17	10	3.7	13
5	F	4.9	20	122	ASD OS	17	0.9	Present	2.1	15	9	MAO	20	12	3.9	13
6	M	49.3	82	171	ASD OS	26	0.3	Absent	2.3	22	12	ASO	30	14	5.2	13
7	M	3.8	16	120	ASD OS, PDA	17	1.1	Present	2.5	18	9	MAO	18	26	7.8	13
8	M	28	91	178	ASD OS	31	0.3	Absent	2.2	17	12	ASO	34	18	5.3	13
9	M	24.3	61	175	ASD OS	21	0.3	Present	2.4	19	12	MAO	24	17	4.6	13
10	M	3.1	18	112	TGA, Arterial switch, residual ASD	12	0.7	Present	2	14	8	ASO	14	9	3.6	10
11	M	10.7	36	127	ASD OS/T21	15	0.4	Present	1.9	14	8	ASO	16	10	3.6	11
12	M	9.8	32	139	ASD OS	19	0.6	Absent	1.8	15	9	ASO	22	14	3.1	10
13	M	14.8	34	137	ASD OS	19	0.6	Present	2.3	18	9	MAO	22	19	6.1	8
14	F	7.6	17	114	ASD OS/T21	19	1.1	Present	2.6	13	10	ASO	22	13	4.2	7
15	F	8	22	123	ASD OS	14	0.6	Present	2.2	17	8	ASO	16	9	3.5	7
16	M	15.1	44	149	ASD OS	24	0.5	Absent	2.6	17	12	FAO	27	8	3.1	7
17	M	10.6	41	143	ASD OS	15	0.4	Absent	1.7	12	7	ASO	16	5	3.3	7

AAI, age at intervention (years); AF, atrial fibrillation; ASD, atrial septal defect; ASO, Amplatzer septal occluder; AWR, ASD size-to-weight ratio; CHD, congenital heart disease; F, female; FAO, Flex II ASD Occluder; MAO, MemoPart ASD Occluder; FU, follow-up (months); MPAP, mean pulmonary artery pressure (mmHg); PDA, patent ductus arteriosus; PT, procedural time (min); Qp/Qs, pulmonary-to-systemic flow ratio; FT, fluoroscopic time (min); M, male; OS, ostium secundum; SS, sheath size (Fr); TGA, transposition of great arteries; T21, trisomy 21; W/H, weight (kg)/height (cm).

### 3.2. Cardiac catheterization

The median size of the non-stretched ASD on two-dimensional ultrasound was 19 mm (IQR, 16–22). Six (35.3%) patients had large ASDs (≥20 mm), three (17.6%) had an ASD size-to-body weight ratio higher than 0.9, five (29.4%) had absent aortic rims, and two (11.8%) had elevated pulmonary arterial pressures. In three patients (nos. 5, 10, and 16), the left retention disc was partially pulled out of the sheath to align the center of the device with the atrial septum. All interventions were successful and straightforward without any complications using different occluder devices (Table 1). The maneuver was successful from the first attempt in all patients with the left disc properly clamping the left atrial wall (Supplementary Videos S1–S3). In patient no. 13, a 20-mm MemoPart ASD Occluder was unstable and was uneventfully upsized to a 22 mm to ensure complete closure. The median device size was 22 mm (IQR, 17–24). The median device size-to-ASD static two-dimensional diameter ratio was 1.14 (IQR, 1.07–1.16), and the median difference between the device size and the ASD two-dimensional static diameter was 3 mm (IQR, 1–3). The median device size-to-body weight ratio was 0.6 (IQR, 0.4–0.8). The median procedural time was 12 min (IQR, 10–14), and the median fluoroscopy time was 4.1 min (IQR, 3.6–4.6). In patient no. 14, a biplane fluoroscopy was used only for demonstration purposes.

### 3.3. Postoperative evaluation and follow-up

All patients were discharged the next postoperative day. Postoperative cardiac ultrasound showed complete shunt closure. No pericardial effusion or bulky device appearance was detected. On a median follow-up of 13 months (IQR, 8–13), no complications were detected. All patients achieved full clinical recovery with complete shunt closure.

## 4. Discussion

Transcatheter ASD closure is considered a complex intervention in case of multiple implantation attempts or when advanced technical maneuvers are needed to achieve successful defect closure (4–6). A significant proportion of ASDs are neither small nor central, and these are more of a challenge for device implantation. Deficiencies of the anterosuperior septum are common, especially in large defects. The usual approach from the inferior caval vein means that the occluder device will approach the atrial septum at a certain angle. Sometimes, it can be challenging to prevent the anterosuperior rim of the device from pulling from the LA into the right atrium before the core of the device can be developed. The ability of interventionists to tackle difficult anatomies from the femoral vein has improved over time with substantial modifications in the techniques of device delivery and deployment (5–12). Modified deployment maneuvers included changing the orientation of the left atrial disc within the LA or adjusting the deployment sequence by delivering the central core of the device slightly within the LA before bridging the device back to the septum and in case of failure delivering the left disc within the left or right upper PV. More advanced deployment maneuvers included the use of modified or steerable delivery sheaths and guidewire-assisted, dilator-assisted, snare-assisted, and/or balloon-assisted closures (7–11). However, all these maneuvers are associated with longer procedural time, higher radiation exposure, and an increased risk of potential complications (12, 13).

We came up with the FAST technique when attempting the left upper PV deployment technique for closing a challenging ASD. However, the device did not open in the PV but in the LA, and the implantation worked with rapid device unsheathing. The utility and advantages of the FAST technique were evident, and we started applying this technique when judged useful. FAST technique turned out to be a simple and safe maneuver for ASD closure with higher chances of device anchorage. The space occupation difficulties are reduced, and the probability that the occluder falls off or shifts is limited. Once the tip of the sheath is in the mid-LA in the direction of the left upper PV and the center of the device is at the atrial septum, the sudden FAST move will deploy both discs together, clamping on the defect almost simultaneously (Supplementary Videos S1–S3). In this case series, we showed that the FAST technique can be easily used independent of the type of occluder device, and all procedures were straightforward. Despite the reported advantages of the newer devices with flexible delivery systems, we believe that the flexibility of the delivery cable has no impact on this rapid single-move maneuver. The major advantage of the flexible delivery systems is the pre-release ultrasound assessment of the device's accommodation over the margins and the presence of a residual leak (15–17). For the FAST technique, fluoroscopic guidance is mandatory to align the center of the device with the atrial septum. Ultrasound also plays a crucial role in tracking the position of the device during the FAST deployment sequence. Considering the 2–3 min duration needed for the baseline hemodynamic catheterization, the median procedural time was only 12 min (IQR, 10–14). Therefore, we believe that this new FAST technique has similar technical descriptions to the left upper PV deployment method. However, it might be safer and superior because it eliminates the risks of PV damage and bleeding (12, 13).

It may take some time to comfortably switch from the usual device implantation method to this FAST technique. Interventionists might need to rapidly adapt to the manipulation of the delivery sheath and the device. The slow uncovering of the left atrial disc and opposing it to the atrial septum are the main causes of longer procedural time after repetitive failed attempts of implantation. Gradual sheath removal to uncover the rest of the device is another pitfall of the classical implantation method. This slow movement allows the left part of the device to change its orientation within the LA and slip into the right atrium before the rest of the device has developed and sandwiched the atrial hole for the right side. Importantly, the smoothness of the sudden unsheathing movement may not be optimal when the maximum device size per sheath size is used. We observed that the manipulation is slightly less smooth and slower (Supplementary Video S10). This might lead to the opening of the left disc completely before releasing the right disc simultaneously and might cause a pull of the left disc on the septal wall. This will be a concern in patients with absent aortic rims because the left disc might prolapse to the right atrium. We suggest upsizing the sheath to the next size when you select a device with the maximum size per sheath size.

Oversizing of the device along with other risk factors has been associated with early and late cardiac erosions (14, 18). However, it is also important to note that not all patients who had oversized devices developed erosion and that erosion has been seen in patients who did not have oversized devices placed (18). There is still a lack of uniform practice when it comes to how interventionists choose device sizes when closing ASDs (18–20). In self-centering devices, the recommended device size is the same or slightly larger than the balloon-sized diameter (20). However, it is also believed that ultrasound-guided sizing of ASD and device deployment provide a better success rate with relatively smaller-sized devices (17, 21). As per our usual experience, we did not balloon-size the defects to avoid unintentional overstretching of the defect diameters. The size of the devices was selected based on the size of the ASDs in diameters as measured on two-dimensional ultrasound. We also avoided device oversizing by keeping the device size-to-ASD static two-dimensional diameter ratio close to 1 and the difference between the median device size and ASD static two-dimensional diameter to ≤4 mm. We did not observe any echocardiographic indicator of a high risk of erosion (18). There was no pericardial effusion on postoperative ultrasounds or significant splaying of the device edges by the aorta in patients with absent aortic rims.

### 4.1. Limitations

This study is conducted with a relatively small number of enrolled patients. However, we believe that our preliminary results of this novel technique should be disseminated to the community. The selection of the device in our series was based only on the availability in the armamentarium. The deployment technique was not compared to other techniques of implantation in terms of procedure and radiation exposure, yet the straightforward and rapid maneuvers to stably position the device support our claims. Application of this FAST implantation method was not tested for the jugular approach. When the defect is addressed from above, the perfectly perpendicular orientation of the delivery sheath to the atrial septum may be easily lost, and the tip of the sheath will be pointing toward the mitral valve. The transjugular safety of this technique must be first confirmed because this rapid maneuver might engage and damage the mitral valve.

## 5. Conclusions

We are presenting a novel FAST technique to safely close secundum ASDs with reduced procedural time and radiation exposure. We find this technique useful to overcome the common problem of left disc malalignment to the septum in defects with absent aortic rims and/or high ASD size-to-body weight ratio. We acknowledge that the LA size could be a limiting factor in case larger defects are encountered in small-sized LAs.

## Data availability statement

The raw data supporting the conclusions of this article will be made available by the authors, upon request, to any qualified researcher.

## Ethics statement

The studies involving human participants were reviewed and approved by the Institutional Review Board. Written informed consent to participate in this study was provided by the participants’ legal guardian/next of kin.

## Supplementary material

Supplementary Video S1 Patient no. 15. Anteroposterior fluoroscopic view of FAST technique. The device is aligned at the atrial septum just right to the mid-spinal line. Both left and right retention discs are rapidly unsheathed, in one single move. The sudden move will deploy both discs together to let them clamp on both sides of the defect almost simultaneously.

Supplementary Video S2 Patient no. 14. Anteroposterior (left panel) and lateral (right panel) fluoroscopic view of FAST technique.

Supplementary Video S3 Patient no. 14. Anteroposterior fluoroscopic view of easier position re-adjustment of the well-oriented device without the anterosuperior rim of the device slipping into the right atrium.

Supplementary Video S4 Patient no. 16. Anteroposterior fluoroscopic view showing the left atrial disk of the Flex II ASD Occluder partially protruded out of the delivery sheath to form a tiny bulb in the LA. This maneuver helps in aligning the center of the device with the atrial septum before proceeding to the FAST technique.

Supplementary Video S5 Patient no. 15. Device stability check.

Supplementary Video S6 Patient no. 15. Device release.

Supplementary Video S7 Patient no. 14. Anteroposterior (left panel) and lateral (right panel) fluoroscopic view of device stability check.

Supplementary Video S8 Patient no. 14. Anteroposterior (left panel) and lateral (right panel) fluoroscopic view of device release.

Supplementary Video S9 Patient no. 16. Device stability check before release.

Supplementary Video S10 Patient no. 2. Anteroposterior fluoroscopic view showing a less smooth and slower unsheathing movement, where the maximum device size per sheath size is used.
